# Interneurons: Role in Maintaining and Restoring Synaptic Plasticity

**DOI:** 10.3389/fpsyt.2016.00086

**Published:** 2016-05-12

**Authors:** Maria Elisa Calcagnotto

**Affiliations:** ^1^Neurophysiology and Neurochemistry of Neuronal Excitability and Synaptic Plasticity Laboratory, Biochemistry Department, Universidade Federal do Rio Grande do Sul, Porto Alegre, Brazil

**Keywords:** GABA, interneurons, synaptic plasticity, brain oscillations, cell replacement

Inhibitory circuits play an important role in synaptic plasticity during development and adulthood. Changes in interneuronal activity induce structural and synaptic rearrangements of inhibitory interneurons, network oscillations, and homeostatic plasticity. In addition to epileptic seizures, deficits in the inhibitory system lead to aberrant information processing and cognitive impairment in various neurological disorders. Studies exploring the structural and functional plasticity of interneurons are essential, not only to understand the mechanisms underlying normal development and behavior but also, to identify the etiology of different psychiatric and neurological disorders to pursue new therapies. Here, it will be discussed the role of inhibitory circuit in the synaptic plasticity, and how cellular replacement strategies can remodel changes in circuit function and homeostasis in the context of brain repair.

## Interneuron Diversity and Origin

Most of the gamma-aminobutyric acid-containing (GABAergic) interneurons in the cerebral cortex and hippocampus originate from three progenitor regions in the embryonic subpallium: caudal ganglionic eminence (CGE), medial ganglionic eminence (MGE), and preoptic area (POA) (1–4). Each progenitor region produces a particular group of interneurons, although some interneuron classes may emerge from different progenitor domains. In the cortex and in the hippocampus, the MGE produces most of the interneurons including fast spiking-parvalbumin (FS-PV)-expressing basket and chandelier cells and somatostatin (SOM)-expressing interneurons with or without coexpression of calretinin (CR), neuropeptide-Y (NPY), or reelin. CGE generates cholecystokinin (CCK), CR, vasointestinal peptide (VIP), reelin, and neurogliaform cells, but not SOM-expressing interneurons. Some interneurons coexpress CR and VIP, whereas others coexpress NPY and reelin. POA originates a small population of reelin and/or NPY-expressing neurons. Recent studies indicate that this region may also give rise to a small fraction of PV- and SOM-expressing cortical interneurons whose development does not depend on Lhx6 function (3). These inhibitory interneurons play key roles in regulating local circuit activity and synaptic plasticity (5).

## Interneurons Orchestrating Synaptic Plasticity and Oscillations

In the cortex and hippocampus, interneurons subtypes differ in their functional connectivity and generate differentially timed inhibition at distinct sites of postsynaptic cells (6–8). Interneurons are perfectly positioned to synchronize network activity. Some target dendritic domains (e.g., SOM-, NPY-, or CB-expressing interneurons) to control the efficacy and plasticity of excitatory inputs onto principal neurons (7). In CA1 hippocampal region, the oriens-lacunosum moleculare (O-LM) and bistratified interneurons, both expressing PV and SOM, are dendritic targeting cells (9). Others target perisomatic compartments (i.e., soma, axon initial segment, and thick proximal dendrites) (e.g., FS-PV-expressing basket cells or CCK-expressing interneurons) (6) to control the output and consequently synchronize the firing rate of principal cells action potentials (10–12). The hippocampal CA1 pyramidal cells soma receive abundant GABAergic inputs from basket cells (13) that are able to control the ability of inputs to generate action potential and to synchronize the pyramidal neurons firing rate (10). Chandelier cells target the axon initial segments of several pyramidal neurons and also contribute to the output synchronization of CA1 principal cells (14). In the mature cortex, a single basket interneuron is able to form characteristic perisomatic synapses (15) with hundreds of pyramidal neurons (16). Each pyramidal neuron, in its turn, can receive inputs from multiple basket cells (16, 17). The postnatal maturation of perisomatic innervation is essential to synchronize pyramidal neurons activity within cortical circuits. In addition to perisomatic inhibition, other FS-PV-expressing interneurons also innervate either axons (chandelier cells) (18) or dendrites (O-LM and bistratified cells) of target cells (7, 9, 19, 20). All these GABAergic inputs in specific subcellular domains play an important role in brain oscillations and plasticity. In particular, the FS-PV-expressing interneurons are known to be crucial in gamma (30–80 Hz) oscillations in the cortex and hippocampus (5, 21, 22), involved in cognition and information processing (23). Accordingly, the blockage of synaptic output of hippocampal PV-expressing interneurons impairs spatial working memory (24). Moreover, the O-LM and bistratified cells that fire in phase with theta oscillations generate global dendritic inhibition, mediate network-state-dependent inhibition on specific parts of pyramidal neuron dendrites, are targeted by afferents from the medial septal region, and are crucial for hippocampal rhythm generation in behaving animal (9, 19, 20). During gamma oscillations, bistratified cells seem to participate in the transmission of the CA3-dependent gamma component to CA1 (19, 20).

Besides the direct control of inhibition, synchrony and plasticity on principal cells, different interneuron subtypes exhibit a wide range of responses to different neuromodulatory inputs, leading to changes in net inhibition, synchronization, and synaptic plasticity (25). The innervation pattern of O-LM cells, which includes the excitation by fast cholinergic transmission, the inhibition of distal dendrites, and the disinhibition of proximal dendrites of pyramidal neurons in CA1, enables them to modulate synaptic efficiency and plasticity of entorhinal cortex and CA3 inputs (19).

Interneuron subtypes differ in their functional connectivity to the principal cells in cortex and hippocampus (6, 8), providing different functional outcome for action potential generation from principal cells (7). This can be exemplified by an elegant work from Ledri and colleagues, where the rupture of hypersynchronization was achieved, by controlling the activity of large populations of interneurons rather than a single population of PV- or SOM-expressing interneurons in the hippocampus, using optogenetics (12). The inhibition of PV-expressing interneurons target by optogenetics also seems to suppress gamma power, while its stimulation elicited gamma oscillations in downstream pyramidal neurons (22), therefore controlling network oscillation.

As mentioned above, GABAergic interneurons targeting dendritic domains control the efficacy and plasticity of excitatory inputs onto principal neurons. This dendritic remodeling of inhibitory neurons, either in normal or pathological conditions, affects activity-dependent modulation of neuronal connectivity within local circuits (26). Reduction in dendritic ramification and decreased axonal length has been described in interneurons in schizophrenia (27) and animal models of epilepsy (28). However, decrease in density and substantial increase in size, axonal reorganization, and aberrant synaptic connections of remaining and newborn SOM-expressing interneurons were observed in hippocampal CA1 and dentate gyrus of animal models of epilepsy (29, 30). This reorganized circuitry synchronizes granule cells activity and decreases seizure threshold, despite the increment in number of GABAergic terminals, contributing to the inhibitory dysfunction in epilepsy. It seems to be an abundant but dysfunctional attempt to compensate the decreased inhibitory input to granule cells after epileptogenic injuries (29, 30). Thus, changes in the synaptic reorganization of SOM-expressing interneurons can disrupt network organization and increase excitation levels.

Together with the interneuronal network connectivity at different cell domains, modulatory inputs and dendritic remodeling, the intrinsic properties of interneurons are particularly crucial to generate and control neuronal oscillations and plasticity (31). For example, the intrinsic properties of FS-PV-expressing interneurons and fast GABA_A_ receptor kinetics are likely to be needed to achieve precise timing gamma oscillations in cortex and hippocampus (21, 22). Additionally, different GABAergic interneuron subtypes fire independently and innervate distinct postsynaptic domains at different time points. These coordinated synaptic interactions actively orchestrate the precise input/output information to generate and control neuronal activity during network oscillations in different developmental and behavioral states (8).

## Interneuron Replacement Restores Synaptic Plasticity and Network Oscillation

As we discussed earlier, GABA transmission has an important role in synaptic plasticity. Moreover, it is essential to regulate plasticity during critical periods of brain development. Changes in inhibition create an environment with aberrant neuronal network that is associated with neurodevelopmental (29, 30, 32–35). Interestingly, some of the most consistent findings in autism spectrum disorders, schizophrenia, epilepsy, and cognitive disorders consist in SOM- and PV-expressing interneurons dysfunction in the brain, including O-LM cells (29, 30, 36–39). Thus, by controlling GABAergic activity, it could be possible to maintain or rescue normal network oscillations and synaptic plasticity.

To address this issue, cell replacement using MGE precursors (source of PV- and SOM-expressing interneurons) has been performed in animal models. The studies had shown that MGE-derived cells are able to survive, differentiate in mature interneurons, migrate, and functionally integrate through the host brain parenchyma with low risk of promoting brain tumors. The integrated mature interneurons modify the neuronal network, rescuing the normal functional inhibition and the synaptic plasticity (40–44). Some examples are studies using MGE precursors grafted into brain of neonate distal less homeobox 1-deficient mice (*Dlx1*^−/−^) (34) and cyclin D2 knockout mice (*Ccnd2*^−/−^) (43). *Dlx1*^−/−^ mice exhibit late-onset interneuron loss and reduced inhibition (34), with consequent deficit in interneuronal network, altered gamma frequency oscillations (GFOs), and dysfunction in homeostatic plasticity and seizures (44). The authors demonstrated that MGE-derived cells reduced seizure severity, restored inhibition, normalize gamma oscillations, and reversed the homeostatic changes in excitatory synaptic activity hippocampal long-term potentiation (44). In the *Ccnd2*^−/−^ mice that display deficit in PV-expressing interneurons, hippocampal disinhibition, increased ventral tegmental area dopamine neuronal activity, and cognitive impairment, the MGE-grafted cells were able to differentiate in long-range survival GABAergic interneurons distributed through the hippocampus and reverse the psychosis and cognitive phenotypes (43).

The maturation of GABAergic interneurons, specifically PV- and SOM-expressing subtypes, has been strongly implicated in critical period of plasticity, such as the development of visual cortex (45, 46). It has been shown that PV- and SOM-expressing interneurons, derived from MGE-precursors grafted into neonate brain, were able to induce ocular dominance plasticity shortly after the normal critical period. Inhibitory-grafted neurons reorganize the cortical circuitry by introducing a new set of weak inhibitory synapses (47), rather than simply enhancing the endogenous mature inhibitory synaptic strength. This pattern of numerous and weak connections is consistent with the form of developing inhibition during the critical period (48). However, suppression of PV-expressing neurons in visual cortex in adult mice also induced plasticity and beyond critical period (49). Therefore, new plasticity can be induced when inhibitory precursors were grafted into visual cortex during development and in adult mice (50–52). The plasticity process seems to be a direct result of modification in neural circuit induced by PV- and SOM-expressing cells integrated into the primary visual cortex. Some suggested mechanism are increased connectivity; increased expression of GABA_A_ receptors containing the α1 subunit, abundant at synapses mediated by PV-expressing interneurons (46); and changes in maturation of interneurons (47, 51).

Changes in GABAergic function mediated by PV- and SOM-expressing interneurons impair synaptic plasticity and disrupt network organization and normal brain oscillations. However, by temporal and selective coordination of interneuronal activity, it is possible to modulate interneuronal network and increase GABA release on different subcellular domains of target cells to maintain or rescue normal network oscillations and synaptic plasticity. The investigation of PV- and SOM-expressing interneurons, using strategies that modify interneuronal network and regulate inhibitory input/output of such interneurons at different time points, as selective target them or using precursor cells grafts, are helping us to better understand the role of these specific interneuron subtypes in controlling and restoring synaptic plasticity and brain oscillations during development and adulthood.

## Author Contributions

The author confirms being the sole contributor of this work and approved it for publication.

## Conflict of Interest Statement

The author declares that the research was conducted in the absence of any commercial or financial relationships that could be construed as a potential conflict of interest.
